# RANKL Impairs the TLR4 Pathway by Increasing TRAF6 and RANK Interaction in Macrophages

**DOI:** 10.1155/2022/7740079

**Published:** 2022-04-12

**Authors:** Ryerson Fonseca Mota, Paulo Henrique Cavalcanti de Araújo, Maria Eduarda Ramos Cezine, Flávia Sayuri Matsuo, Rodrigo Jair Morandi Metzner, Carlos Alberto Oliveira de Biagi Junior, Kamila Chagas Peronni, Hiroki Hayashi, Munehisa Shimamura, Hironori Nakagami, Mariana Kiomy Osako

**Affiliations:** ^1^Department of Cell and Molecular Biology, Ribeirao Preto Medical School, University of Sao Paulo, Ribeirao Preto, Sao Paulo, Brazil; ^2^Institute for Cancer Research, Guarapuava, Parana, Brazil; ^3^Department of Health Development and Medicine, Osaka University Graduate School of Medicine, Suita, Osaka, Japan

## Abstract

High serum levels of osteoprotegerin (OPG) are found in patients with obesity, type 2 diabetes, sepsis, or septic shock and are associated with a high mortality rate in stroke. The primary known function of OPG is to bind to the receptor activator of NF-*κ*B ligand (RANKL), and by doing so, it inhibits the binding between RANKL and its receptor (RANK). TLR4 signaling in macrophages involves TRAF6 recruitment and contributes to low-grade chronic inflammation in adipose tissue. LPS is a classical activator of the TLR4 pathway and induces the expression of inflammatory cytokines in macrophages. We have previously observed that in the presence of RANKL, there is no LPS-induced activation of TLR4 in macrophages. In this study, we investigated the crosstalk between RANK and TLR4 pathways in macrophages stimulated with both RANKL and LPS to unveil the role of OPG in inflammatory processes. We found that RANKL inhibits TLR4 activation by binding to RANK, promoting the binding between TRAF6 and RANK, lowering TLR4 activation and the expression of proinflammatory mediators. Furthermore, high OPG levels aggravate inflammation by inhibiting RANKL. Our findings elect RANKL as a candidate for drug development as a way to mitigate the impact of obesity-induced inflammation in patients.

## 1. Introduction

The adipose tissue is an essential endocrine organ that has a central role in obesity-associated complications such as dyslipidemia, insulin resistance, and type 2 diabetes. In obese patients, the adipose tissue consists of adipocytes, preadipocytes, vascular, neural, and immune cells—such as macrophages [1, 2]. These cells secrete a wide range of hormones and cytokines, including tumor necrosis factor-alfa (TNF-*α*), interleukin-1 beta (IL-1*β*), and macrophage chemoattractant protein- (MCP-) 1, which induces macrophage infiltration and low-grade inflammation that eventually results in a global impairment of glucose metabolism and insulin resistance [3].

Osteoprotegerin (OPG) is a soluble protein that lacks transmembrane and cytoplasmic domains. High serum OPG levels have been found in patients with obesity [4] and type 2 diabetes [5], but whether OPG high levels were deleterious or beneficial is unclear.

A previous study reported that patients with sepsis or in septic shock have higher levels of OPG when compared to the controls [6]. Additionally, patients with higher levels of OPG when entering the intensive care unit have a higher risk of dying in the first 30 days than patients with lower levels of OPG [7]. High serum OPG level is also associated with a high mortality rate in patients with stroke [8, 9].

The primary biological function of OPG is to bind to the receptor activator of the NF-*κ*B ligand (RANKL) [10]. We have recently reported that RANKL induces differentiation of beige adipocytes responsible for increasing energy expenditure and improving glucose metabolism in white adipose tissue [11]. However, the role of RANKL in macrophages infiltrated in adipose tissue has not been explored yet.

Our previous study has shown that RANKL lowers neuronal death rates induced by inflammatory cytokines released by microglia during the ischemic stroke [12]. These data suggest that RANKL may have anti-inflammatory properties, but the molecular mechanism is still elusive.

Macrophages can be activated by interferon-gamma (IFN-*γ*) and lipopolysaccharide (LPS, a component of the cell wall of gram-negative bacteria) to express IL-1*β*, TNF-*α*, and inducible nitric oxide synthase (iNOS, which generates reactive NO species with microbicide and proinflammatory properties) [13, 14]. Macrophage activation by LPS involves the toll-like receptor 4 (TLR4) [15], an essential pattern recognition receptor involved in the innate immune system response. Upon TLR4 binding to LPS, its cytosolic domain interacts with several adaptor proteins, such as TNF-receptor-associated factor 6 (TRAF6), resulting in activation of IKK and phosphorylation of the I*κ*B*α*, its subsequent ubiquitination, degradation, and release of NF-*κ*B. This transcription factor, in turn, activates the transcription of proinflammatory mediators [16].

TLR4 can also be activated by endogenous molecules released from dying or lysed cells [17]. High-fat diet-induced hypertrophied adipocytes eventually undergo cell lysis in the adipose tissue, releasing large amounts of free fatty acids. These free fatty acids can activate innate immune cells via TLR4 expressed in macrophages, which leads to proinflammatory cytokine expression and adipose tissue inflammation [18]. Pretreatment with RANKL reduces the cytokine storm in LPS-induced septic shock in a murine model by decreasing the expression of adaptor proteins of the TLR4 pathway, such as myeloid differentiation factor 88 (MyD88), IL-1 receptor associated-kinase 1 (IRAK1), and TRAF6 [19]. The latter is an adaptor protein shared by both pathways (RANK and TLR4) [16, 20].

Preliminary observations done by our group suggest that, in the presence of RANKL, there is no LPS-induced activation of TLR4 in macrophages, even though the RNA expression of the adaptor proteins (MyD88, IRAK1, and TRAF6) does not change when compared to the group not submitted to RANKL stimulation (unpublished data). This novel observation led us to raise the hypothesis that an additional mechanism might inhibit activation of the TLR4 pathway in macrophages.

Considering that the primary known function of OPG is to bind to RANKL and by doing that OPG inhibits the binding between RANKL and its receptor (RANK), in this study, we investigated the crosstalk between RANK and TLR4 pathways in macrophages costimulated with RANKL and LPS, to unveil the role of OPG in inflammatory processes.

## 2. Material and Methods

### 2.1. Animal Studies

Animal care and experimentation were performed following the Ethical Principles in Animal Research adopted by the National Council for the Control of Animal Experimentation (CONCEA), and this study was approved under the protocol number 047/2017 by the Animal Care and Use Committee of Ribeirao Preto Medical School of University of Sao Paulo.

The wild-type C57BL/6J (WT) and the B6.129S4-Tnfrsf11b tm1Eac/J (OPG-/-) mice were obtained from Jackson Laboratories. All animals used in this study were 8-week-old male mice. Standard diet (ND) consisted of AIN-93G diet, and the high-fat diet (HFD) consisted of AIN-93G modified to contain 35% lipids (4% soy oil and 31% lard). All mice had access to water and food *ad libitum* and were housed in the animal facility at 20°C-22°C under a 12 h light-dark cycle.

### 2.2. Histological Analysis

The dissected adipose tissue consisted of visceral white adipose tissue (vWAT) located in the epididymal region and fixed and prepared for histological analysis. Paraffin sections were stained with hematoxylin and eosin (H&E), and the images were taken by Scanscope (Olympus BX61VS).

### 2.3. *Ex Vivo* Culture

The *ex vivo* explant culture was performed as described before [21]. Visceral white adipose tissues were dissected, washed with PBS, and supplemented with penicillin (100 U/ml) and streptomycin (100 *μ*g/ml), cut in small pieces, weighted, distributed in 24-well plates, and cultured in RPMI. The supernatant was collected after 24 h, centrifuged, and used as adipose tissue-conditioned medium (ATCM) or centrifuged and stored at -80°C until ELISA assays were performed.

### 2.4. Enzyme-Linked Immunosorbent Assay (ELISA)

IL-1*β* and TNF-*α* levels were measured using the 96-well strip plate Quantikine mouse IL-1beta ELISA Kit (R&D Systems, MLB00C) and TNF-alfa ELISA Kit (R&D Systems, MTA00B), respectively.

### 2.5. Cell Culture

Immortalized monocyte lineages obtained from patients with acute monocytic leukemia (THP-1 cells) from Rio de Janeiro Cell Bank (#0234) were cultivated with RPMI supplemented with 10% of fetal bovine serum (FBS) and penicillin (100 U/ml) and streptomycin (100 *μ*g/ml). The THP-1 cells were seeded at a confluence of 1 × 10^6^ cells on 3.5 cm diameter plates and derived into macrophages by stimulation with PMA (5 ng/ml) for 24 h. Subsequently, THP1-derived macrophages were stimulated with recombinant RANKL protein (Peprotech) at 10 ng/ml, LPS (Sigma Aldrich) at 10 ng/ml, or costimulus LPS+RANKL.

### 2.6. Colocalization Analysis by Immunofluorescence Microscopy

Anti-RANK (H-300, #sc-9072, Santa Cruz Biotechnologies) and anti-TRAF6 (D-10, #sc-8409, Santa Cruz Biotechnologies) were used to label macrophages with their respective secondary antibodies, Alexa Fluor 488 anti-rabbit (#A21042, Invitrogen), and Alexa Fluor 594 anti-mouse (# A21125, Invitrogen). Immunofluorescence images were acquired with a Leica TCS-SP5 confocal microscope (Leica Microsystems). The images were obtained as confocal stacks of ~60 optical sections of 1, 024 × 1,024 pixels. Each cell stack was used to generate a colocalization value with background correction, performed in triplicate, and quantified as described before [22]. Fiji (ImageJ) was used for image processing, and Manders' correlation coefficient was used to calculate colocalization on each cell section.

### 2.7. RT-Quantitative PCR

RNA was extracted from cultured cells with Tri Reagent (Sigma Aldrich) and reverse-transcribed using High Capacity cDNA Reverse Transcription Kit (Applied Biosystems). The resulting cDNA was analyzed by qPCR using QuantiFast SYBR Green (Qiagen). Reactions were performed in 96-well plate format using ABI PRISM 7500 instrument (Applied Biosystems), and relative mRNA levels were calculated through comparative CT normalized to glyceraldehyde-3-phosphate dehydrogenase (GAPDH) expression in each sample. Gene expression data was presented on a logarithmic scale. The sequences of the primer sets were as follows: IL-1*β* forward CTCACCTCTCCTACTCACTT and reverse CGGTTGCTCATCAGAATGT; TNF-*α* forward CCTGGGATTCAGGAATCTC and reverse GTCAGGGATCAAAAGCTGTAG; iNOS forward ACAAGCCTACCCCTCCAGAT and reverse CTTGGATGGTTGACTGCCCT; and GAPDH forward GGTCTCCTCTGACTTCAACA and reverse CCACAGGAAATGAGCTTGAC.

### 2.8. Western Blot

Whole-cell extracts were prepared in RIPA buffer containing protease inhibitor cocktail (Roche). The total protein concentration was determined with *DC* Protein Assay (Bio-Rad Laboratories). Equivalent doses of proteins were then size-fractionated in 10% SDS-PAGE, transferred to a 0.45 *μ*m pore size PVDF Immobilon-P membrane (Millipore), subjected to 5% BSA blockage, and then incubated with the following antibodies: TLR4 (#ab183459, Abcam), RANK (#ab200369, Abcam), TRAF6 (#ab33915, Abcam), and *β*-actin (#sc-81178, Santa Cruz Biotechnologies. The bound antibodies were detected by the respective horseradish peroxidase-conjugated anti-IgG antibody (Amersham Biosciences) followed by ECL detection system (Amersham Biosciences) according to the manufacturer's instructions.

### 2.9. Co-Immunoprecipitation Assays

Whole-cell extracts were prepared in RIPA lysis buffer containing protease inhibitor cocktail (Roche), and lysis was reinforced using a syringe with a 0.33 × 13 mm needle. The total protein concentration was determined with *DC* Protein Assay (Bio-Rad Laboratories). An equivalent amount of proteins (750 *μ*g/sample) was incubated with a rabbit primary antibody anti-TRAF6 (#ab33915, Abcam) for 3 h at 4°C. Streptavidin magnetic sepharose beads (#GE28, GE Healthcare Life Sciences) were incubated with a biotin-conjugated anti-rabbit IgG antibody (#ab64257, Abcam) for 2 h at 4°C. After washing, magnetic beads were mixed with the proteins, incubated with the primary antibody overnight at 4°C, and eluted. The purified protein concentration was determined with *DC* Protein Assay (Bio-Rad Laboratories) and followed with western blot assays (2 *μ*g/well) to analyze TRAF6 interaction with RANK and TLR4.

### 2.10. Transfection and Luciferase Assays

A population of 1, 5 × 10^6^ THP-1-derived macrophages were seeded on a 3.5 cm plate and transfected using Lipofectamine® 2000 (2 *μ*l/ml) (Invitrogen) diluted in Opti-MEM (Gibco) with 5 *μ*g of the luciferase reporter plasmid regulated by NF-*κ*B, pNF*κ*B-Luc (#631745, Clontech) or a plasmid expressing human RANK point mutated, pcDN3A-human-RANK∆, in a final volume of 1 ml per plate. The cells were transfected for 5 h, and the medium was replaced with 1 ml of supplemented RPMI. After 24 h, the cells were stimulated with recombinant RANKL, LPS, or costimulus LPS+RANKL for 24 h. The pcDN3A-human-RANK*∆* plasmid was constructed by using a pcDNA3 template and insertion of the human RANK sequence (NCBI GenBank AF018253.1) point mutated at TRAF6 binding sites, 344-349 PTEDEY, 377-382 PLEVGE, and 453-458 PGEDHE by the substitution of glutamic acid (E) for alanine (A) in residues 346, 379, and 455. Luciferase activity was quantified accordingly to the manufacturer's instructions (Promega). Briefly, the cells were collected and lysed, and an equivalent protein concentration was incubated in the presence of luciferin substrate. The luminescence was measured using a 96-well plate, and data were shown as CPS—counts per second.

### 2.11. RNA Isolation from Bone Marrow-Derived Macrophages

Bone marrow-derived macrophages were collected from the femur and tibia of wild-type mice and cultivated in DMEM supplemented with 10% FBS, 100 U/ml penicillin, 100 *μ*g/ml streptomycin, and macrophage colony-stimulating factor (M-CSF, 20 ng/ml). After 7 days, the attached cells were stimulated with RANKL (10 ng/ml) for 24 h. RNA was extracted from cultured cells with Tri Reagent (Sigma), RNA quality was checked with an Agilent TapeStation (Agilent Technologies, Inc.), and all RINs were between 7.7 and 9.1.

### 2.12. Library Preparation and Deep Sequencing

Library preparation was performed according to the manufacturer's protocol using the Illumina Stranded mRNA Sample Preparation Kit. Briefly, the extracted RNA was quantified using Qubit assays (Life Technologies, USA) to ensure an adequate RNA concentration (500 ng), followed by polyA mRNA selection and fragmentation, first and second strand synthesis, adenylation of 3′ ends, and index adapter ligation. Each library was subjected to 15 cycles of PCR amplification, and the size distribution was examined on an Agilent TapeStation (Agilent Technologies, Inc.) using a D1000 ScreenTape. All libraries displayed a band between 200 and 500 bp with a peak at approximately 330 bp. The libraries were quantified with Qubit 2.0 Fluorometer (Life Technologies, Inc.), loaded at a concentration of 2.3 nM with 15 libraries, pooled, and sequenced on Illumina NovaSeq Sequencing System with paired-end 150 bp reads.

### 2.13. Mapping of RNA-Seq Libraries and Differential Expression Analysis

The bcl files generated by the sequencer were converted to fastq using the software bcl2fastq (v2.20.0.422). Based on the fastq files, data quality control was performed using FastQC, followed by alignment and mapping of the reads against the reference genome (GRCm38.101) and counting using RSEM software (v1.3.1), and the alignment file was sorted using samtools (v1.11). All individual count files per sample were aggregated, thus having a single count matrix. Differential expression analysis was performed using DESeq2 package (v1.30.1), and the functional enrichment was performed using the clusterProfiler package (v3.18.1). The plots were generated using ggplot2 (v3.3.3) and ComplexHeatmap (2.6.2) packages in R (v4.0.5).

### 2.14. Statistical Analysis

All data were presented as SEM analyzed using GraphPad Prism v. 7.0 (San Diego, CA, USA). Statistical significance was determined by either one-way ANOVA followed by Bonferroni's posttest or unpaired two-tailed Student's *t*-test. Significance presented as *p* < 0.05, *p* < 0.01, and *p* < 0.0001.

## 3. Results

### 3.1. RANKL Reduces Inflammation in Adipose Tissue and Macrophages

**Figure 1:**
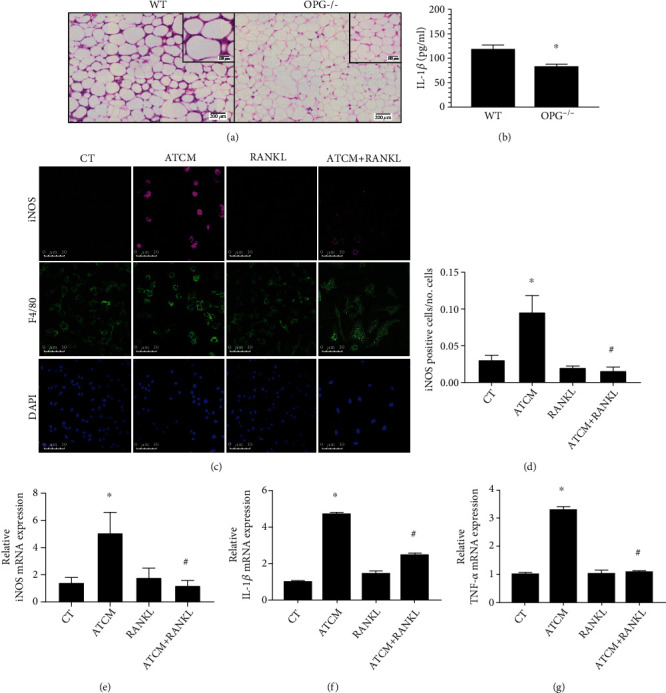
RANKL reduces inflammation in adipose tissue and macrophages. (a) Representative images of H.E.-stained visceral white adipose tissue (vWAT) from wild-type (WT) mice showing increased cell infiltration and crown-like structure formation (inset image) compared to OPG-/- mice under a high-fat diet for 3 months. (b) Interleukin-1*β* (IL-1*β*) level in the supernatant of vWAT from a 3-month high-fat diet-fed WT and OPG-/- mice cultured *ex vivo* for 24 h and detected by ELISA, *n* = 6, ∗*p* < 0.05 vs. WT. (c) Representative images of macrophages immunostained to detect iNOS (magenta), F4/80 (green), and nuclei (DAPI, blue) by immunofluorescence in THP1 macrophages with culture medium (CT), stimulated with RANKL (10 ng/ml), and adipose tissue-conditioned medium (ATCM) with or without RANKL (10 ng/ml) (ATCM+RANKL) for 24 h. (d) Quantification of iNOS positive cells in CT, RANKL, ATCM, and ATCM+RANKL groups. The quantification was based on the threshold measured in 6 fields of each sample by ImageJ software (*n* = 40). (e) Quantification of relative iNOS mRNA expression in ATCM, RANKL, and ATCM+RANKL stimulated THP1 macrophages for 24 h, detected by qPCR, and compared to the nonstimulated control group (CT). (e and f) IL-1*β* and TNF-*α* expression in bone marrow-derived macrophages stimulated with ATCM and RANKL (10 ng/ml) for 24 h detected by qPCR and compared to the nonstimulated control group (CT). ∗*p* < 0.01 vs. CT and #*p* < 0.01*vs.* ATCM. All data are presented as means ± SD.

Adipose tissues from obese animals and humans also show leucocyte infiltration in visceral white adipose tissue (vWAT) due to adipocyte hypertrophy and eventual release of intracellular components during cell lysis [23, 24]. Free fatty acids, for example, are recognized by TLR4 in macrophages that form crown-like structures (CLS) around dying adipocytes and secrete proinflammatory cytokines. However, CLS were not observed in adipose tissue from OPG-/- mice under a high-fat diet for three months when compared to the wild-type (WT) mice (Figure 1(a)). In addition, vWAT explants cultured for 24 h from OPG-/- mice showed a lower production of IL-1*β*, when compared to explants from WT mice (Figure 1(b)). THP1 macrophages stimulated with adipose tissue-conditioned medium (ATCM) showed marked expression of iNOS, a proinflammatory marker, whose level decreased in the presence of RANKL, as seen by immunofluorescence (Figures 1(c) and 1(d)) and quantification of mRNA level (Figure 1(e)). RANKL showed a similar effect in bone marrow-derived macrophages (BMDMs) stimulated with ATCM, as it lowered expression of IL-1*β* and TNF-*α* (Figures 1(f) and 1(g)). These data suggest that RANKL decreases the inflammatory responses in macrophages elicited by secreted factors from hypertrophied adipocytes.

### 3.2. RANKL Lowers LPS Response in Macrophages

**Figure 2:**
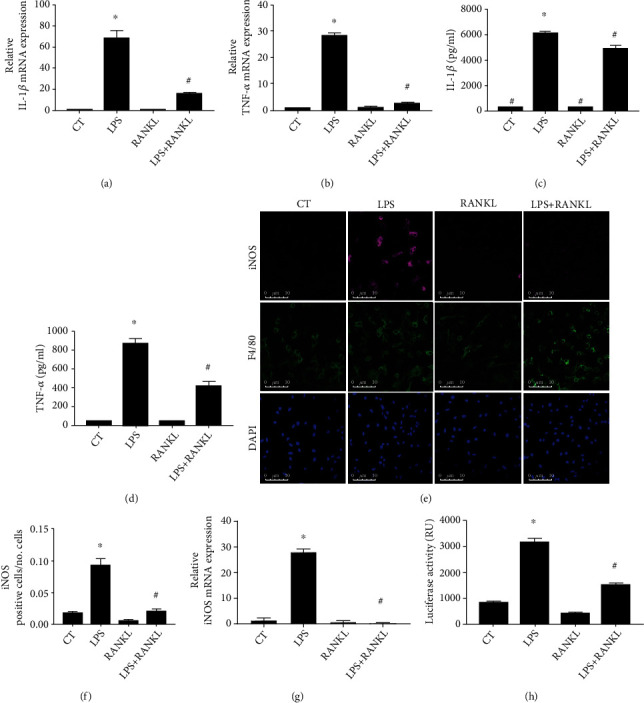
RANKL lowers LPS response in macrophages. (a and b) IL-1*β* and TNF-*α* expression in THP1 macrophages stimulated with LPS (10 ng/ml) and RANKL (10 ng/ml) or costimulated with both (LPS+RANKL) for 24 h, detected by qPCR, and compared to the nonstimulated control group (CT). (c and d) IL-1*β* and TNF-*α* protein level in supernatant of THP1 macrophages stimulated with LPS (10 ng/ml) and RANKL (10 ng/ml) or costimulated with both (LPS+RANKL) for 24 h and detected by ELISA. (e) Representative images of macrophages immunostained to detect iNOS (magenta), F4/80 (green), and nuclei (DAPI, blue) by immunofluorescence in THP1 macrophages stimulated with LPS (10 ng/ml) and RANKL (10 ng/ml) or costimulated with both (LPS+RANKL) for 24 h. (f) Quantification of iNOS positive cells in LPS and RANKL stimulated THP1 macrophages. The quantification was based on the threshold in 6 fields of each sample by ImageJ software (*n* = 4). (g) Quantification of relative iNOS mRNA expression in THP1 macrophages stimulated with LPS and RANKL or costimulated with both (LPS+RANKL) for 24 h, detected by qPCR, and compared to the nonstimulated control group (CT). (h) Quantification of NF-*κ*B activation in THP1 macrophages transfected with a plasmid expressing NF-*κ*B-driven luciferase stimulated with LPS (10 ng/ml) and RANKL (10 ng/ml) or costimulated with both (LPS+RANKL) for 24 h and detected by luciferase activity presented as relative units (RU). A one-way ANOVA followed by Bonferroni's multiple comparisons test was performed to compare all groups (∗*p* < 0.01 vs. CT and #*p* < 0.01 vs. LPS). The data represent the mean of three independent experiments and are presented as means ± SD.

LPS is a classical activator of the TLR4 pathway and induced macrophages into a higher inflammatory cytokine expression profile that included IL-1*β* and TNF-*α* expression, but not when costimulated with RANKL (Figures 2(a)–2(d)). Accordingly, macrophages stimulated with RANKL and LPS showed lower expression of iNOS when compared to the LPS group, as quantified by immunofluorescence (Figures 2(e) and 2(f)) and expression of mRNA level (Figure 2(g)). Activation of TLR4 pathway culminates in I*κ*B*α* phosphorylation (phospho-I*κ*B*α*), which is ubiquitinated, undergoes proteasome degradation, and releases NF*κ*B. Accordingly, THP1 macrophages expressing NF*κ*B-driven luciferase reporter gene showed increased luciferase activity under LPS stimulation, but it decreased under LPS+RANKL costimulation (Figure 2(h)). These data indicate that similar to the response elicited by adipose tissue-conditioned medium, RANKL decreased the LPS-induced response in macrophages, suggesting that RANK activation impairs the TLR4 signaling pathway in a way that anticipates NF-*κ*B activation.

### 3.3. RANKL Promotes TRAF6 and RANK Interaction in Detriment to TLR4 in LPS-Induced Macrophages

**Figure 3:**
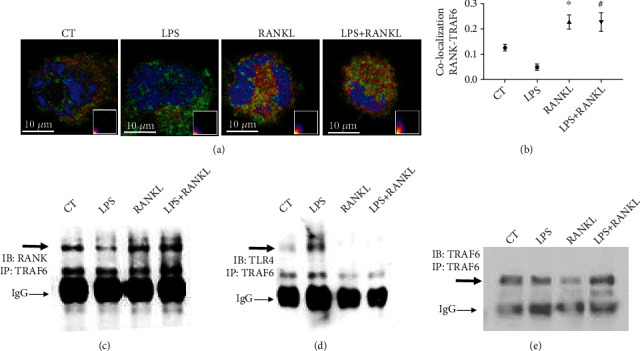
RANKL promotes TRAF6 and RANK interaction in detriment to TLR4 in LPS-induced macrophages. (a) Representative images of RANK and TRAF6 colocalization by confocal microscopy in THP1 macrophages stimulated with LPS (10 ng/ml) and RANKL (10 ng/ml) and costimulated with both (LPS+RANKL) for 10 min or nonstimulated as the control group (CT), inset: colocalization scattergram graphs. The cells were labeled with RANK (green), TRAF6 (red), and DAPI (blue). (b) Quantification of protein interaction by RANK-TRAF6 immunofluorescence colocalization. Manders' correlation coefficient was used to calculate colocalization of the cell sections. A one-way ANOVA followed by Bonferroni's multiple comparisons test was performed to compare all groups, *n* = 6. All data are presented as mean ± SEM. ∗*p* < 0.0001 vs. CT. #*p* < 0.0001 vs. LPS). (c, d, and e) Detection by western blot of RANK, TLR4, and TRAF6 co-immunoprecipitated with TRAF6 in protein extracts from THP1 macrophages stimulated with LPS (10 ng/ml) and RANKL (10 ng/ml) or costimulated with both (LPS+RANKL) for 10 min.

RANKL activates the receptor RANK to elicit a cellular response, a process that depends on TRAF6, which is also involved in the TLR4 activation pathway [25]. Macrophages stimulated with RANKL show marked colocalization of RANK and TRAF6, as shown by immunofluorescence (Figure 3(a)). Interestingly, the colocalization degree was the same in RANKL alone and LPS+RANKL costimulated cells (Figure 3(b)). To confirm an existing interaction between RANK and TRAF6, protein extracts of macrophages stimulated for 10 min with LPS, RANKL, or both were co-immunoprecipitated with a TRAF6 antibody. As expected, RANK coprecipitates with TRAF6 under RANKL stimulation, which is not observed in the group stimulated with LPS only (Figure 3(c)). However, higher RANK levels were bound to TRAF6 in cells under LPS+RANKL costimulation (Figure 3(c)). Conversely, lower levels of TLR4 have bound to TRAF6 in LPS+RANKL costimulated THP1 macrophages if compared to the LPS group (Figure 3(d)) and the total level of TRAF6 (Figure 3(e)). Therefore, TRAF6 binds to RANK under the presence of RANKL and LPS, which impairs the TLR4 pathway activation.

### 3.4. RANKL Inhibits LPS-Induced Cytokine Expression upon TRAF6/RANK Binding

**Figure 4:**
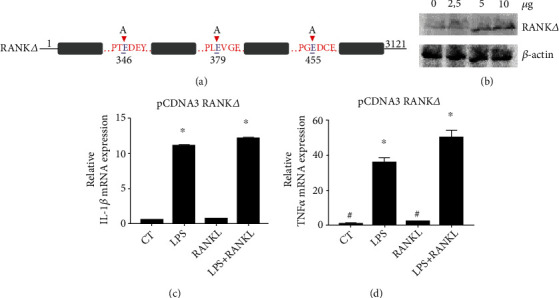
RANKL inhibits LPS-induced cytokine expression upon TRAF6/RANK binding. (a) The sequence of RANK points mutated in 3 different glutamic acid residues in TRAF6 binding sites (RANK*Δ*). (b) Detection of RANK by western blot in THP1 macrophages after transfection of different doses of pCDNA3 plasmid expressing. (c) IL-1*β* and (d) TNF-*α* expression in THP1 macrophages transfected with 5 *μ*g of RANK*Δ*-expressing pCDNA3 plasmid and stimulated with LPS (10 ng/ml) and RANKL (10 ng/ml) and costimulated with both (LPS+RANKL) for 24 h or nonstimulated control group (CT) and detected by qPCR. A one-way ANOVA followed by Bonferroni's multiple comparisons test was performed to compare all groups. All data are presented as mean ± SEM. ∗*p* < 0.0001 vs. CT.

RANK interaction with TRAF6 is essential for RANKL signaling in osteoclasts as TRAF6-/- mice show severe osteopetrosis [20]. As RANK interacts with TRAF6 with 3 domains (residues 344-349, 377-382, and 453-458) [26], a mutated version of RANK (RANK*∆*) was generated by the substitution of glutamic acid (E) for alanine (A) in residues 346, 379, and 455 (Figure 4(a)) and transfected into macrophages (Figure 4(b)) to investigate the relevance of RANK/TRAF6 binding in the anti-inflammatory response of RANKL. The overexpression of RANK*∆* abrogated the effect of RANKL in LPS-induced expression of IL-1*β* and TNF-*α* (Figures 4(c) and 4(d)). Thus, RANK/TRAF6 binding is essential for RANKL inhibition in LPS-induced TLR4 activation.

### 3.5. RANKL Downregulates the Pathways Related to Chemotaxis in BMDM

**Figure 5:**
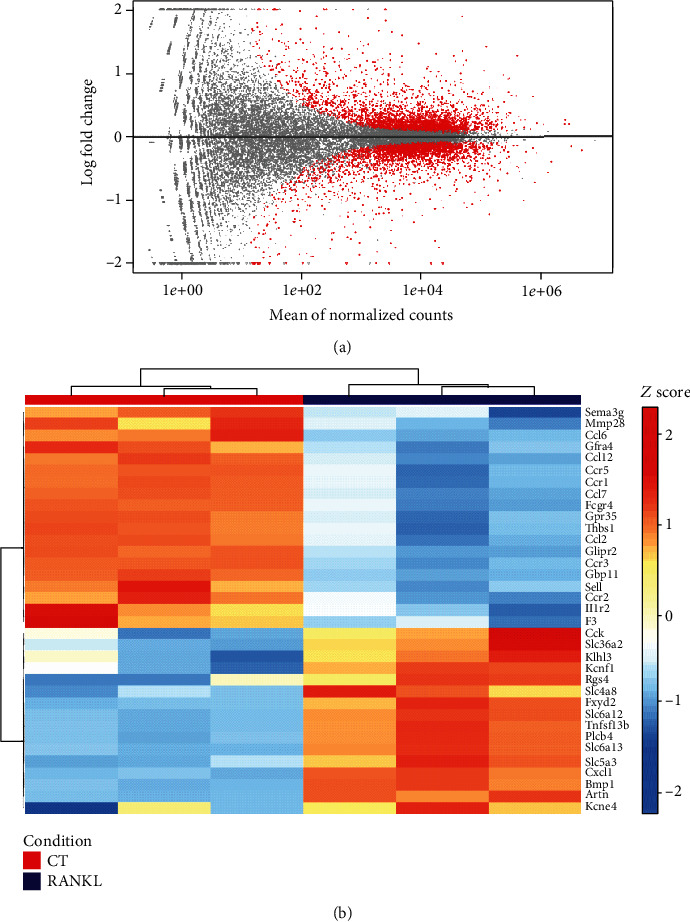
RANKL downregulates the pathways related to chemotaxis in BMDM. (a) MA plot for log2 folds changes values against normalized counts for each gene in the analysis. Red points mark genes with FDR < 0.1. (b) Visualization as a heat map of genes that are up- and downregulated in BMDM without (CT) or with RANKL stimulation (10 ng/ml) (RANKL) for 24 h.

To confirm the global effect of RANKL in inhibiting inflammatory responses in macrophages, BMDMs collected from WT mice and stimulated for 24 h with RANKL showed a differential expression of 376 transcripts (Figure 5(a)). In functional enrichment analysis (Figure 5(b)), RANKL stimulation downregulated the expression of genes related to chemotaxis in proinflammatory macrophages, such as *Ccl6*, *Ccl7*, and *Ccr2*, and upregulated the expression of genes related to inorganic cation transport, such as *Slc4a8* and *Kcnf1* [27, 28] and also downregulated *Bmp1*, a metalloprotease that activates transforming grow factor-*β* [29]. Even though the transcript for *Tnfsf13b*, related to osteoclastogenesis [30], was upregulated by RANKL stimulation, macrophages stimulated with RANKL showed no multicellularity or TRAP activity, markers for osteoclast differentiation (data not shown). Taken together, RANKL stimulation for 24 h downregulates the expression of genes related to chemotaxis of proinflammatory macrophages without inducing osteoclastogenesis.

## 4. Discussion

In the present study, we have shown that RANKL inhibits TLR4 activation in macrophages by binding to RANK, promoting the binding between TRAF6 (crucial for TLR4 activation) and RANK, affecting events occurring upstream of NF-*κ*B activation, and thus lowering the expression of proinflammatory mediators.

High-fat diet-fed WT mice show crown-like structures formed by macrophages surrounding dying adipocytes in visceral adipose tissue [23, 24]. The expression of proinflammatory cytokines in these infiltrated macrophages is increased by TLR4, which is stimulated by intracellular components such as free fatty acids released from hypertrophied adipocytes [31, 32].

In OPG-/- mice, high levels of circulating RANKL are free to bind to RANK, promoting its activation [10, 11]. And we have previously reported that OPG-/- mice show browning of subcutaneous and visceral adipose tissue and that RANKL stimulates beige adipocyte differentiation into beige adipocytes [11]. Moreover, macrophages infiltrated in the visceral adipose tissue of OPG-/- mice under a high-fat diet showed lower expression of iNOS when compared to those of WT mice [11]. In this study, we observed no macrophage infiltration and no crown-like structure formation in visceral adipose tissue of OPG-/- mice under a high-fat diet for 3 months (Figure 1(a)). In accordance with our previous work [11], we found lower levels of IL-1*β* in visceral adipose tissue of OPG-/- mice (Figure 1(b)), which reinforces the observation of lower inflammation in adipose tissue of OPG-/- mice, when compared to controls. Macrophages cultivated in adipose tissue-conditioned medium (ATCM) with RANKL showed lower expression of proinflammatory mediators such as iNOS, IL-1*β*, and TNF-*α* (Figures 1(c)–1(g)). Together, these data suggest that by binding RANK, RANKL reduces inflammation in adipose tissue and macrophages.

The adipose tissue-conditioned medium contains large amounts of saturated fatty acids recognized by TLR4 [33] and many unknown components. LPS is considered the classical activator of TLR4; it elicits a proinflammatory response in macrophages, such as the expression and secretion of critical proinflammatory mediators such as IL-1*β*, TNF-*α*, and iNOS. In response to IL-1*β* binding, a complex sequence of phosphorylation and ubiquitination events leads to activation of NF-*κ*B, JNK, and p38 MAPK pathways, which together induce the expression of target genes such as IL-6, IL-8, and MCP-1, that further amplify the proinflammatory response [34–36]. TNF-*α* binds to the receptor TNFR1, which belongs to a group of death receptors (DR) containing death domains (DD) associated with a cytoplasmic silencer of the death domain (SODD) in the resting state [37]. Upon TNF-*α* activation, SODD is released, and the DD domain of TNFR1 can interact with other adaptor proteins that activate transcription factors such as NF-*κ*B and AP-1, which increase the expression of cytokines, adhesive molecules, and metalloproteinases, for example [38]. LPS stimulation also increases iNOS levels, an enzyme that triggers NO synthesis, which acts as a cytotoxic effector molecule with antimicrobial activity in macrophages and damages normal cells. These proinflammatory mediators released by macrophages upon LPS stimulation are also found in adipose tissue inflammation induced by obesity [39]. In high-fat diet mice, adipose tissue macrophages show increased expression of genes essential for macrophage migration, phagocytosis, proinflammatory cytokines such as IL-6, IL-1*β*, and TNF-*α*, and iNOS [39, 40], which induces a low-grade proinflammatory state. TNF-*α* downregulates perilipin (PLIN) expression, a small protein that coats the intracellular triglyceride lipid droplet in white adipocytes, regulating lipolysis rate and adipocyte fatty acid secretion. Hence, TNF-*α* increases lipolysis in human and lineage adipocytes [41, 42]. In this context, macrophages are recruited by adipocyte components released during lysis of hypertrophied cells, such as free fatty acids, capable of activating TLR4 to produce proinflammatory cytokines in macrophages, which favors TNF-*α*-induced lipolysis and more TLR4 activation [18].

Thus, to confirm the findings observed with adipose tissue-conditioned medium stimulation, macrophages were costimulated with LPS+RANKL to assess the effect of RANKL-induced RANK stimulation on the TLR4 pathway activation. Based on our observation that in the presence of RANKL, expressions of LPS-induced factors TNF-*α*, IL-1*β*, and iNOS are reduced (Figures 2(a)–2(g)), we propose that the anti-inflammatory property of RANKL is through inhibition of TLR4 activation. Macrophages pretreated for 6 h or 24 h with RANKL showed reduced TLR4 activation under LPS stimulation due to lower mRNA expression of adaptor proteins that participate in the TLR4 signaling transduction pathway [19]. Here, macrophages submitted to stimulation with LPS+RANKL showed no difference regarding the expression of the mRNAs of adaptor proteins such as Myd88, IRAK, and TRAF6 (data not shown) after 6 h or 24 h of costimulation, even though we did observe lower expression of IL-1*β* and TNF-*α* during this same time course (Figures 2(a) and 2(b)). Thus, this data indicates that RANKL stimulation inhibited events in the TLR4 pathway occurring upstream of the expression of proinflammatory cytokines.

The TLR4 pathway culminates in the activation of several transcription factors [43]. NF-*κ*B activation is crucial for LPS-induced proinflammatory cytokine expression [44]. In other scenarios, RANKL has been reported to increase NF-*κ*B activation as a proinflammatory response in the liver and pancreas [45]. Even though RANKL is named for receptor activator of NF-*κ*B ligand, RANKL-induced NF-*κ*B activation is not always related to inflammatory cell response. For example, RANKL was first identified in bone and named ODF (osteoclast differentiation factor) [46] because it induces osteoclast activation and bone resorption by activating the transcription factors NF-*κ*B and NFATc [47, 48]. Additionally, RANKL was found to activate RANK (and named for receptor activator of NF*κ*B) in dendritic cells, in which RANKL increases the ability to stimulate T cells [49, 50]. In this study, we show that RANKL does not change IL-1*β*, TNF-*α*, or iNOS expression levels in macrophages nor it significantly changes the NF-*κ*B activation in macrophages, as shown by the luciferase reporter assay (Figure 2(h)).

TRAF6 is a common adaptor protein in both TLR4 and RANK pathways [20, 51, 52] and is activated upstream the NF-*κ*B nuclear translocation in the TLR4 pathway. RANKL stimulation increased colocalization of RANK and TRAF6, as detected by confocal microscopy, in the LPS+RANKL stimulated group when compared to the LPS group (Figures 3(a) and 3(b)). Protein extracts from macrophages treated with RANKL, LPS, and LPS+RANKL were analyzed for endogenous TRAF6 interaction with RANK or TLR4 by co-immunoprecipitation. We observed that RANK co-immunoprecipitated with TRAF6 in cells treated with RANKL and LPS+RANKL, but not in cells stimulated with LPS only (Figure 3(c)). These findings indicate that in the presence of RANKL, TRAF6 binds to RANK, even if LPS is present. LPS treatment promotes the binding between TLR4 and TRAF6, a reason why we observed higher levels of TLR4/TRAF6 co-immunoprecipitation. However, this is not observed when cells are stimulated with LPS+RANKL (Figure 3(d)), given by the presence of TRAF6 in the pull-down (Figure 3(e)). These data indicate that RANKL impairs TLR4 activation by promoting TRAF6 interaction with RANK. These findings indicate that in the presence of LPS, RANKL stimulation promotes TRAF6 interaction with RANK while decreasing TRAF6 interaction with TLR4.

Macrophages overexpressing a version of RANK carrying a point mutation in the domain of TRAF6-binding sites presented similar levels of IL-1*β* and TNF-*α* expression in LPS+RANKL groups as those observed in the LPS group (Figures 4(c) and 4(d)). These findings suggest that RANK interaction with TRAF6 is crucial for RANKL inhibition of TLR4-induced gene expression.

TRAF6 is an ubiquitin E3 ligase that catalyzes lysine-63 (K63) polyubiquitination in target proteins such as I*κ*B kinase or as a self-polyubiquitination of TRAF6, which in turn forms a recognition signal for the recruitment of proteins such as TAB2/3 and TAK1, leading to NF-*κ*B activation. TRAF6 can also be modified by K48-linked ubiquitination that targets proteasome degradation [53–55]. Therefore, future analysis should investigate if RANKL stimulation routes TRAF6 to degradation by increasing TRAF6-K48 ubiquitination in macrophages and blocking the inflammatory process.

Global analysis of gene expression by RNA-seq showed that RANKL stimulation induced a differential expression of 376 transcripts in macrophages (Figure 5(a)), downregulating the expression of genes such as *Ccl6*, *Ccl7*, and *Ccr2*, which are related to chemotaxis of proinflammatory macrophages (Figure 5(b)) [56–58]. Although *Tnfsf13b* was upregulated, which is related to osteoclastogenesis [30], the macrophages stimulated with RANKL showed no osteoclastogenesis (data not shown). This finding indicates that RANKL lowers the expression of target genes involved in chemotaxis besides the impairment of TLR4 activation. Further studies are necessary to clarify how the binding between TRAF6 and RANK results in sustained inhibition of NF-*κ*B activation in macrophages.

It should be noted that the experiments performed here were restricted to THP1 macrophages, immortalized human monocyte, and BMDMs from wild-type mice. In addition, the effect of RANKL was not evaluated in macrophages in inflammatory contexts in animals or patients. Therefore, the interpretation of these results may be limited, and future studies are warranted in human macrophages under a chronic low-grade inflammatory state found in obesity or acute inflammation such as septic shock, for example.

Infection with severe acute respiratory syndrome coronavirus 2 (SARS-CoV-2) that developed into coronavirus disease 2019 (COVID-19) can lead to respiratory failure and death in patients. A growing body of clinical studies suggests that a cytokine storm triggered by the viral infection is associated with COVID-19 severity and is also a crucial cause of death in this disease [59, 60]. Retrospective studies have shown that obesity is an independent risk factor for complications in COVID-19, and it suggests that the chronic inflammation induced in the adipose tissue can increase the risk for cytokine storm [61–63]. Thus, RANKL could be a potential candidate for drug development to mitigate the impact of obesity-induced inflammation in patients.

## 5. Conclusions

This study shows for the first time that RANKL increases TRAF6 interaction with RANK to the detriment of TLR4 and decreases proinflammatory responses in macrophages. It also provides new insights on the role of RANKL in inflammatory processes in macrophages elicited by TLR4 activation and suggests that high OPG levels found in obese patients or under septic shock could be considered a poor prognostic in part because OPG may aggravate inflammation by inhibiting RANKL.

## Data Availability

Data is available under request to the corresponding author (Mariana Kiomy Osako, mko@fmrp.usp.br).
